# Cognitive Impairment and the correlation with genetic Expression of GAD67, Gad65 and GABA beta2 Using Human Induced Pluripotent Stem Cells

**DOI:** 10.1192/j.eurpsy.2022.801

**Published:** 2022-09-01

**Authors:** D. Khalifa, H. Gabr, H. Fathy, H. Abdou, M. Batrawy

**Affiliations:** 1 Kasr Al Ainy hospitals, Cairo University, Psychiatry, Cairo, Egypt; 2 Kasr Al Ainy hospitals, Cairo University, Clinical Pathology, Cairo, Egypt

**Keywords:** GAD67, Genetic expression, schizophrénia

## Abstract

**Introduction:**

Alteration of GABergic neurotransmission is accused to be sharing in the cognitive impairment in schizophrenia. Exploring the relation between the neuronal expression of GABergic genes and cognitive impairment in living patients through modeling of schizophrenia is an important step to know more about the core of the pathophysiology of this disorder

**Objectives:**

Altered genetic expression of GAD 67 may have an important role in the pathophysiology of cognitive impairment in schizophrenia

**Methods:**

. Reprogramming of human fibroblasts into human induced pluripotent stem cells (hIPSc) then neuronal differentiation was performed in 20 patients presenting with schizophrenia and 20 matched controls. Real time Polymerase chain reaction was done for measurement of genetic expression of GAD 65, GAD 67 and GABA beta 2. The Digit Symbol task, block design, block design task and similarities tasks from the Wechsler Adult Intelligence Scale., Trail A and Trail B making tests in addition to Rey-Osterrieth Complex Figure Test (ROCF) were applied to measure cognitive functions .

**Results:**

There were lower means of GAD65, GAD67 and GABA beta2genetic expression in the patients group with significant statistical difference between the 2 groups. The down regulation of GAD 67 in patients presenting with schizophrenia is positively correlated with impairment in executive functions.

**Conclusions:**

GAD 67 gene expression had the most significant correlations with the cognitive assessment in both patients and controls. The presence of those statistically significant correlations in both groups points to the possible role of GAD 67 gene functioning in the pathophysiology of cognitive impairment in schizophrenia

**Disclosure:**

No significant relationships.

